# Clinical acceptance testing and scanner comparison of ultrasound shear wave elastography

**DOI:** 10.1002/acm2.12310

**Published:** 2018-03-15

**Authors:** Zaiyang Long, Donald J. Tradup, Pengfei Song, Scott F. Stekel, Shigao Chen, Katrina N. Glazebrook, Nicholas J. Hangiandreou

**Affiliations:** ^1^ Department of Radiology Mayo Clinic Rochester MN USA

**Keywords:** acceptance testing, GE, shear wave elastography, Supersonic, target measurements, ultrasound

## Abstract

Because of the rapidly growing use of ultrasound shear wave elastography (SWE) in clinical practices, there is a significant need for development of clinical physics performance assessment methods for this technology. This study aims to report two clinical medical physicists’ tasks: (a) acceptance testing (AT) of SWE function on ten commercial ultrasound systems for clinical liver application and (b) comparison of SWE measurements of targets across vendors for clinical musculoskeletal application. For AT, ten GE LOGIQ E9 XDclear 2.0 scanners with ten C1‐6‐D and ten 9L‐D transducers were studied using two commercial homogenous phantoms. Five measurements were acquired at two depths for each scanner/transducer pair by two operators. Additional tests were performed to access effects of different coupling media, phantom locations and operators. System deviations were less than 5% of group mean or three times standard deviation; therefore, all systems passed AT. A test protocol was provided based on results that no statistically significant difference was observed between using ultrasound gel and salt water for coupling, among different phantom locations, and that interoperator and intraoperator coefficient of variation was less than 3%. For SWE target measurements, two systems were compared — a Supersonic Aixplorer scanner with a SL10‐2 and a SL15‐4 transducer, and an abovementioned GE scanner with 9L‐D transducer. Two stepped cylinders with diameters of 4.05–10.40 mm were measured both longitudinally and transaxially. Target shear wave speed quantification was performed using an in‐house MATLAB program. Using the target shear wave speed deduced from phantom specs as a reference, SL15‐4 performed the best at the measured depth. However, it was challenging to reliably measure a 4.05 mm target for either system. The reported test methods and results could provide important information when dealing with SWE‐related tasks in the clinical environment.

## 1 INTRODUCTION

Ultrasound elasticity imaging is one of the most noteworthy technologies developed in ultrasound imaging in the last two decades. Extensive research efforts have been devoted to the development of various methods, such as compression elastography, transient shear wave imaging, acoustic radiation force imaging, crawling wave imaging, etc.^1^, ^2^, ^3^ These methods may also be classified according to the excitation approaches, i.e., the quasi‐static or dynamic methods. Despite the variations, the general idea is to perturb tissue with external or internal mechanical sources to generate a measurable displacement, detect the axial or shear deformation, and then deduce a parameter that is related to tissue elasticity. Either qualitative or quantitative assessment can be achieved. A method is deemed to be quantitative if Young's modulus or shear modulus, or shear wave speed can be directly determined.

Some of these methods, imaging or nonimaging, point or 2D measurements, qualitative or quantitative, have been realized on commercial ultrasound scanners from several vendors.^4^, ^5^ Among them, shear wave elastography (SWE) recently has become available on multiple systems and is attractive due to its 2D imaging capability and quantitative nature. Because of the unique tissue mechanical information provided, numerous clinical studies have demonstrated the clinical utility of ultrasound elastography, such as in liver, breast, prostate, kidney, pancreas, and musculoskeletal (MSK) applications.^6^, ^7^, ^8^, ^9^, ^10^, ^11^ For example, 2D SWE was shown to have high sensitivity in the assessment of liver fibrosis.^12^ The utility of SWE was also demonstrated in assessing the elasticity of normal and pathologic Achilles tendon.^13^, ^14^

Because of ultrasound's accessible and affordable nature, many clinical practices are looking into the possibility of providing ultrasound SWE service. Clinical medical physicists are naturally involved in the process of bringing ultrasound elastography into clinical practice. For example, we have faced tasks to perform an acceptance testing (AT) of multiple scanner systems for their SWE function for liver applications, as well as to evaluate different vendors for potential MSK applications of target measurements such as nerves and soft tissue lesions. Despite the intense research developments and clinical applications of SWE functionality itself, few publications exist on the clinical physics aspects of assessing ultrasound elastography performance.^15^ In addition, there is no regulatory requirement on acceptance testing or quality assurance on SWE currently. The aim of this work was to report our acceptance testing results and the recommended protocol, as well as our evaluation methods and results of target SWE measurements using systems across vendors.

## 2 MATERIALS AND METHODS

### 2.A Acceptance testing using homogeneous phantoms

Ten new GE LOGIQ E9 XDclear 2.0 scanners (GE Healthcare, Milwaukee, WI, USA), each equipped with a C1‐6‐D and a 9L‐D transducer, were purchased by our practice and included in this study. All scanners and transducers had undergone acceptance testing for physical and mechanical integrity, uniformity, geometric accuracy, depth of penetration, contrast response, and spatial resolution. All systems passed our acceptance testing. The SWE function on these systems was realized by implementation of the comb‐push technology with directional filtering and time‐interleaved interpolation of the shear wave tracking, to overcome the lack of software beamforming and low tracking pulse repetition frequency on conventional ultrasound scanners.^16^ For AT, we decided to test the performance consistency among the ten systems instead of absolute accuracy, because there appears to be a lack of gold standard in phantom material measurement.^17^ Two of the Model 039 shear wave liver fibrosis phantoms were chosen as the test objects (CIRS Inc., Norfolk, VA, USA) (“soft phantom” and “hard phantom” with reference Young's moduli of 3 and 45 kPa, respectively). The reference shear wave speed of the targets can be calculated to be 0.985 and 3.816 m/s using the following equation, assuming a phantom density of 1.03 g/cm^3^ as shown in the phantom specification using the following equation. $E=3\rho {c_{s}}^{2},$ where *E* is the Young's modulus, ρ is the material density, and *c*_s_ is the shear wave speed.

Two experienced operators measured the shear wave speed using each of the transducers on its scanner with the two phantoms. Two imaging depths were obtained (1 and 4 cm for 9L‐D, 3 and 7 cm for C1‐6‐D), because of the known depth dependency of shear wave speed measurements.^18^ A circular region of interest (ROI) with an area of 1.8 cm^2^ was centered at the abovementioned depths. The average shear wave speed from the circular ROI was recorded. Five repeats were obtained for each measurement. Salt water with a concentration of 4.5 g sodium chloride (Sigma‐Aldrich, St. Louis, MO, USA) per 100 mL degassed water was used for coupling, as suggested by the RSNA Quantitative Imaging Biomarker Alliance (QIBA) shear wave speed biomarker committee.^19^ Transducers were placed in contact with the phantom surface without additional pressure. Acquisition parameters were kept the same among all systems, i.e., push output 100%, track output 100%, shear wave vibration frequency 150–400 Hz for C1‐6‐D, and 100–500 Hz for 9L‐D. All measurements were performed using phantom mode.

Mean and standard deviation (SD) of shear wave speed measurements among all systems are reported. System deviation was assessed as a percentage of the group mean, (max operator (|group min – group mean|, |group max – group mean|))/group mean × 100. It was also assessed as multiples of the group SD, (max operator (|group min – group mean|, |group max – group mean|))/SD. System deviation from the group mean value that was less than either 5% or 3SD was deemed to be acceptable in our AT. Related‐samples Wilcoxon signed rank test was used to compare the corresponding system shear wave speeds at different depths.

**Figure 1:**
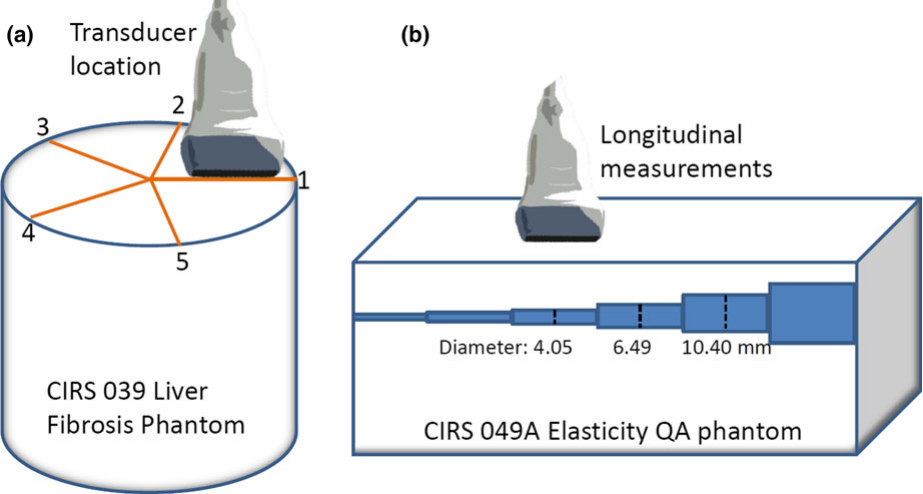
Schematic representations of the experiment setup for (a) investigating the effects of different coupling media, operators, and phantom locations (homogeneity) for the acceptance testing protocol, and (b) longitudinal measurements of the cylindrical test objects for small target task.

In addition, we also studied several aspects of our AT methodology. In order to assess effects of different coupling media, operators, and phantom locations (homogeneity), additional experiments were performed by marking five locations on the surface of the hard phantom [Fig. 1(a)]. Three operators, two abovementioned and another experienced operator, performed five measurements at each of the five marked locations at the same two imaging depths using salt water or a generous amount of ultrasound gel (Cardinal Health, Waukegan, IL, USA) for good coupling. One operator acquired the same dataset after 1 week. To assess whether there was a difference due to the coupling medium, paired sample *t*‐test was used to compare the corresponding 40 sets of measurements made with ultrasound gel and salt water, among all operators and depths. To assess operator variations, interoperator coefficient of variation (CV) was calculated among all three operators. Intraoperator CV was also calculated within one operator at two time points. To determine whether different phantom locations should be considered in the testing, phantom location deviation was calculated as for each location, (max operator (|group min – group mean|, |group max – group mean|))/group mean × 100%.

### Comparison of target SWE measurements across vendors

A Supersonic Aixplorer ultrasound scanner (Supersonic Imagine, Aix‐en‐Provence, France) equipped with an SL10‐2 and SL15‐4 transducer and a GE LOGIQ E9 XDclear 2.0 scanner equipped with a 9L‐D transducer were compared for the task of measuring shear wave speed in small targets such as tendons and nerves. The Aixplorer scanner utilizes multiple acoustic radiation force push beams applied at increasing focal depths to form coherently added shear waves in a cone shape, and monitor shear wave propagation across a field of view using plane waves at thousands of frames per second.^20^ To evaluate clinical applications of SWE measurement of tendons and nerves, the model 049A elasticity QA phantom (CIRS Inc., Norfolk, VA, USA) was chosen for this task because of its stepped cylindrical targets surrounded in a homogeneous background (background reference Young's modulus 25 kPa, reference shear wave speed 2.844 m/s). Two of the stepped cylinders (“soft target” and “hard target” with reference Young's moduli of 8 and 45 kPa, reference shear wave speeds of 1.609 and 3.816 m/s, respectively) with diameters of 10.40, 6.49, and 4.05 mm were selected for this task. The center of these targets was at a depth of 3 cm.

Both transaxial and longitudinal measurements were acquired with five repeats for each target on each scanner/transducer pair [Fig. 1(b)]. Acquisitions parameters were based on scanner defaults and kept consistent, i.e., standard SWE optimization, smoothing 5, persistence high, shear wave frequency 70–680 Hz for SL10‐2 and 70–800 Hz for SL15‐4. GE 9L‐D was set the same as in acceptance testing. To make consistent and precise measurement of targets, a custom program was written to locate the target based on the corresponding gray‐scale image and extract shear wave speed information (MATLAB R2013b, MathWorks Inc., Natick, MA, USA), instead of user‐drawn ROI on the scanner. The sizes of the ROI were based on the known target dimension minus four pixels for transaxial measurements. For longitudinal measurements, ROI length was 50 pixels wide. Each pixel value in the ROI was mapped to the color scale with known speed scale to extract the shear wave speed. All transaxial and longitudinal shear wave speed measurements are reported. In addition, to account for difficulties in accurately specifying mechanical properties of phantom materials,^17^ the value of the hard phantom divided by the value of the soft phantom was used as a reference ratio (3.816/1.609 = 2.37). The corresponding ratio values from our measurements were also calculated and the value closest to the reference ratio was deemed to be the most accurate. The corresponding ratios among all three scanner/transducers were compared using the related‐samples Friedman's two‐way analysis of variance by ranks.

## 3 RESULTS

### 3.A Acceptance testing using homogeneous phantoms

Tables 1 and 2 demonstrate the AT results among all GE systems using the two homogenous phantoms. For systems with C1‐6‐D transducers, the maximum system deviation was 1.6% (2.3 SD) with the soft phantom at the deeper depth, and 5.3% (2.0 SD) for the hard phantom at the deeper depth. For systems with 9L‐D transducers, the max system deviation was 5.4% (2.3 SD) with the soft phantom at the deeper depth, and 3.7% (2.0 SD) for the hard phantom at the deeper depth.

**Table 1:** Mean and standard deviation (SD) of the shear wave speed measurements (m/s) in the acceptance test of ten GE LOGIQ E9 XDclear 2.0 systems. Shallower and deeper depths were 1 and 4 cm, and 3 and 7 cm, for the 9L‐D and C1‐6‐D transducers, respectively

	Soft phantom		Hard phantom	
	Shallower depth	Deeper depth	Shallower depth	Deeper depth
Systems with C1‐6‐D transducers	0.97 ± 0.01	1.00 ± 0.01	3.74 ± 0.03	3.83 ± 0.10
Systems with 9L‐D transducers	0.98 ± 0.01	0.97 ± 0.02	3.56 ± 0.02	3.66 ± 0.07

**Table 2:** Maximum deviation of any individual shear wave speed measurement from the group mean in the acceptance test of ten GE LOGIQ E9 XDclear 2.0 systems (expressed as percentages of the group mean and as multiples of the standard deviation (SD)). Shallower and deeper depths were 1 and 4 cm, and 3 and 7 cm, for the 9L‐D and C1‐6‐D transducers, respectively

	Soft phantom		Hard phantom	
	Shallower depth	Deeper depth	Shallower depth	Deeper depth
Systems with C1‐6‐D transducers	1.2% (1.5 SD)	1.6% (2.3 SD)	1.4% (1.5 SD)	5.3% (2.0 SD)
Systems with 9L‐D transducers	1.3% (2.2 SD)	5.4% (2.3 SD)	1.1% (2.0 SD)	3.7% (2.0 SD)

Among all GE systems with C1‐6‐D transducers, shear wave speeds were 0.97 ± 0.01 and 1.00 ± 0.01 m/s at 3 cm and 7 cm in the soft phantom, respectively. There was a statistical significant difference between them (*P* < 0.01). Shear wave speeds were 3.74 ± 0.03 and 3.83 ± 0.10 m/s at 3 and 7 cm in the hard phantom, respectively. There was also a statistical significant difference between them (*P* < 0.05). A statistically significant difference in shear wave speed measurements was also observed at the two depths in the hard phantom for 9L‐D transducers (*P* < 0.01).

**Figure 2:**
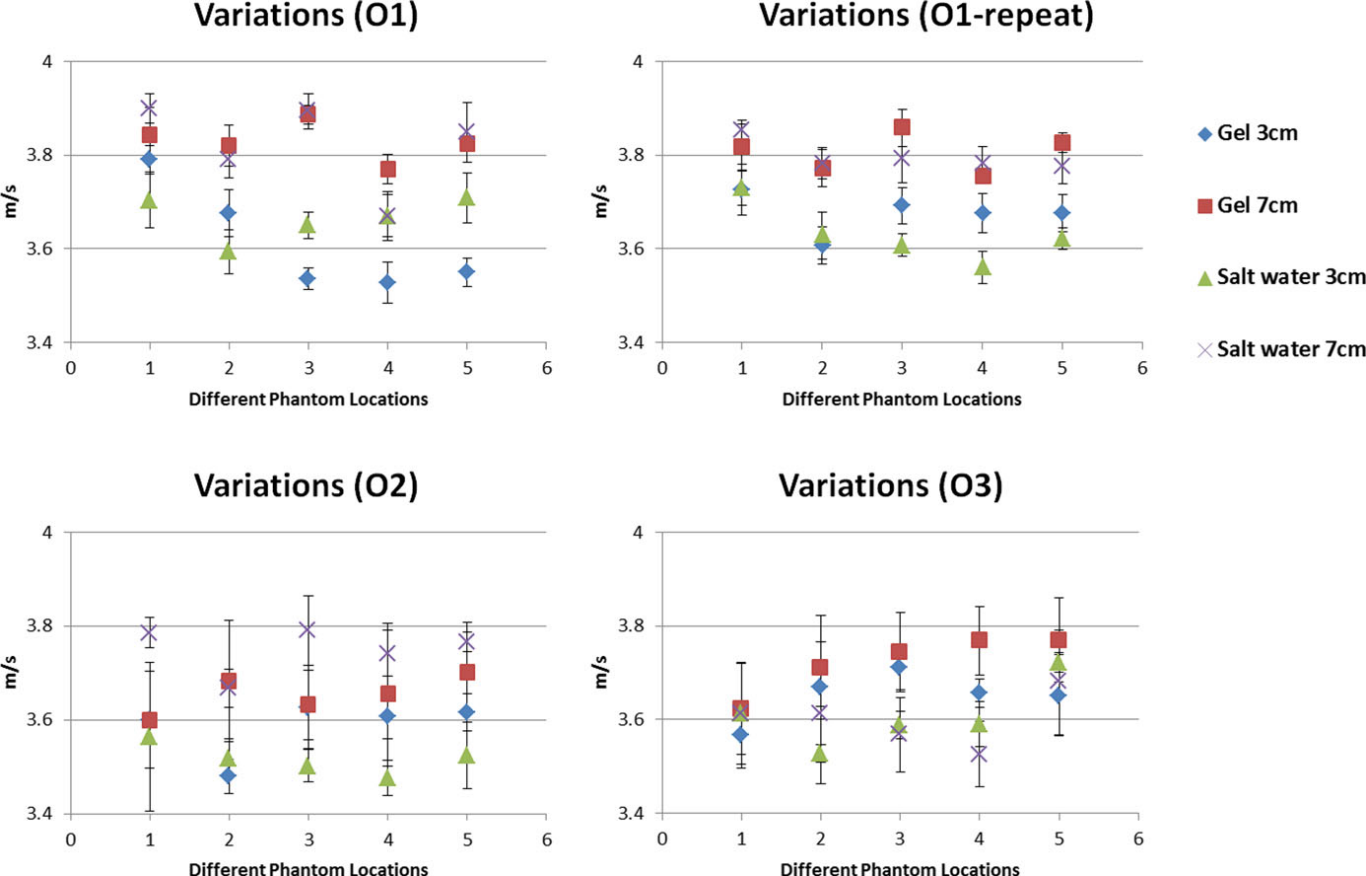
Shear wave speed measurements made by using different coupling mediums (ultrasound gel or salt water), three operators (O1, O2, and O3), and five fixed probe locations in the homogeneous phantom, in order to assess these factors for the acceptance testing protocol. Operator 1 (O1) also repeated the same dataset after 1 week (O1‐repeat).

In addition, Fig. 2 depicts the shear wave speed measurements made by using different coupling mediums (ultrasound gel or salt water), three operators, and five fixed probe locations on the phantom. No difference in shear wave speed measurements was observed between using salt water and ultrasound gel for coupling (*P* = 0.54). Interoperator CV was 2.4% among all three operators and intraoperator CV was 1.6% within one operator between the two sessions separated by a week. Maximum deviation in phantom locations was 2.4% in the hard phantom among five locations and two depths.

### 3.B Comparison of target measurements across vendors

**Figure 3:**
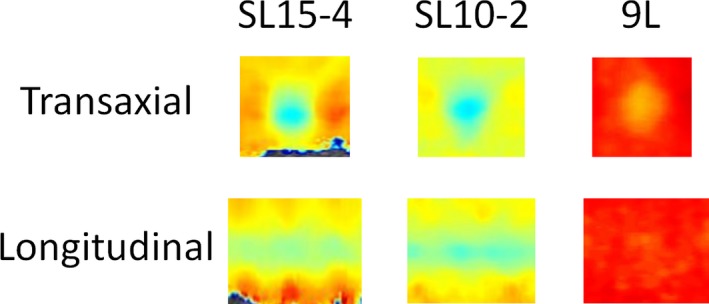
Sample images of transaxial and longitudinal shear wave speed for the 4.05 mm soft target using the Supersonic Aixplorer scanner with SL15‐4 and SL10‐2 transducers, as well as the GE LOGIQ E9 XDclear 2.0 scanner with the 9L‐D transducer.

**Figure 4:**
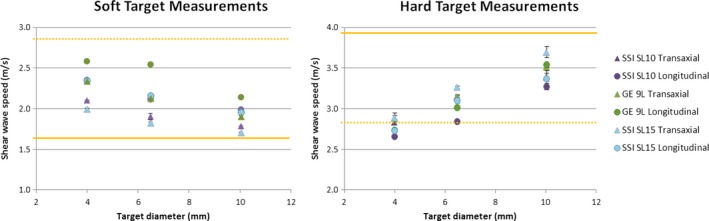
Transaxial and longitudinal measurements of shear wave speed of the soft and hard targets with three diameters using all scanner/transducer pairs. The reference shear wave speed of the targets is indicated with solid lines and that of the background is indicated with dashed lines.

Figure 3 shows sample images for each scanner/transducer pair for the 4.05 mm soft target in both longitudinal and transaxial views. Quantitative measurements of all system/transducer pairs for all targets in different diameters are depicted in Fig. 4, with the reference shear wave speed values of the targets (solid lines) and background (dashed lines). Transaxial and longitudinal measurements are shown in Tables 3 and 4, respectively. A statistically significant difference was revealed among all corresponding shear wave speed ratios among SL15‐4, SL10‐2, and 9L‐D transducers (*P* < 0.01) (Table 5). SL15‐4 transaxial measurement of the 10.4 mm target showed the closest match to the reference shear wave speed ratio between the hard and soft targets.

**Table 3:** Transaxial shear wave speed measurements (m/s) of all targets of different diameters using the Supersonic Aixplorer scanner with the SL10‐2 and SL15‐4 transducers, as well as the GE LOGIQ E9 scanner with the 9L‐D transducer

	Soft target			Hard target		
	10.40 mm	6.49 mm	4.05 mm	10.40 mm	6.49 mm	4.05 mm
Supersonic SL10‐2	1.78 ± 0.01	1.90 ± 0.04	2.10 ± 0.01	3.39 ± 0.05	3.14 ± 0.03	2.83 ± 0.01
Supersonic SL15‐4	1.70 ± 0.01	1.82 ± 0.01	1.99 ± 0.02	3.70 ± 0.07	3.26 ± 0.02	2.89 ± 0.02
GE 9L‐D	1.90 ± 0.01	2.12 ± 0.01	2.33 ± 0.02	3.52 ± 0.01	3.15 ± 0.01	2.88 ± 0.06

**Table 4:** Longitudinal shear wave speed measurements (m/s) of all targets of different diameters using the Supersonic Aixplorer scanner with the SL10‐2 and SL15‐4 transducers, as well as the GE LOGIQ E9 scanner with the 9L‐D transducer

	Soft target			Hard target		
	10.40 mm	6.49 mm	4.05 mm	10.40 mm	6.49 mm	4.05 mm
Supersonic SL10‐2	1.99 ± 0.01	2.11 ± 0.004	2.35 ± 0.003	3.27 ± 0.04	2.84 ± 0.01	2.66 ± 0.01
Supersonic SL15‐4	1.95 ± 0.01	2.16 ± 0.01	2.34 ± 0.003	3.37 ± 0.10	3.10 ± 0.03	2.73 ± 0.01
GE 9L‐D	2.14 ± 0.01	2.54 ± 0.02	2.58 ± 0.01	3.54 ± 0.01	3.01 ± 0.02	2.74 ± 0.01

**Table 5:** Shear wave speed ratios between the hard and soft targets from all scanner/transducer pair measurements and all target diameters. Both transaxial and longitudinal planes are included. The reference ratio from the phantom specification was 2.37

	Transaxial			Longitudinal		
	10.40 mm	6.49 mm	4.05 mm	10.40 mm	6.49 mm	4.05 mm
Supersonic SL10‐2	1.90	1.65	1.35	1.64	1.35	1.13
Supersonic SL15‐4	2.18	1.79	1.45	1.73	1.44	1.17
GE 9L‐D	1.85	1.49	1.24	1.65	1.19	1.06

## 4 DISCUSSION

In this study, we conducted an AT of ten GE LOGIQ E9 XDclear 2.0 systems for liver applications and compared target shear wave measurements of two systems from different vendors. These tasks could be faced by many clinical imaging physicists nowadays. Systemic differences were shown among different transducers and vendors, which were similarly observed in the literature.^18^, ^21^, ^22^

For the acceptance tests, all system deviations from group mean were within 5% or 3 SD. Therefore, all systems passed the AT and were accepted for clinical use. Moreover, no statistically significant difference was found between using ultrasound gel or salt water for coupling. Therefore, the AT protocol was updated to not limit to salt water for coupling. Ultrasound gel is used for clinical patients and is more readily available compared to the abovementioned salt water. Interoperator and intraoperator CV values were both small (<3%). Therefore, the number of operators or exact test timing does not need to be strictly controlled, as long as the operators have general knowledge of SWE function. Considering the largest deviations for each transducer and each phantom occurred at the deeper depths, the AT protocol could be limited to measurements at one depth, i.e., a deeper depth. This would allow shortening of the exam time while still capturing the system variation. In addition, measurements among different phantom locations showed small variations (all <3%). Therefore, phantom locations do not need to be specifically controlled for the specific commercial phantoms used in this work. Overall, the updated AT protocol incorporating these options will maximize the test flexibility and efficiency without compromising the test results.

For comparison of scanners of target measurements, differences in shear wave speed measurements between the two systems were also observed. There are known intrinsic differences in the hardware and software designs between the GE LOGIQ E9 and the Supersonic Aixplorer systems studied here.^16^, ^20^ The Aixplorer scanner with the SL15‐4 transducer performed the best for target measurements using the reference shear wave speed ratio between hard and soft targets. The transaxial measurement of the largest target (10.4 mm diameter) was the closest to the reference shear wave speed ratio. This ratio became smaller with smaller target diameters, when using longitudinal plane, or for lower frequency transducers for all scanner/transducer pairs. It should be noted that penetration of the SL15‐4 transducer was limited as one would expect (Fig. 4). In addition, band‐like artifact regions in the axial direction were also noted on the Aixplorer scanner,^23^ especially in the longitudinal target images. This might be related to the pushing pulses.^23^ These artifacts were avoided when making all ROI measurements.

Our results also clearly demonstrated that longitudinal measurements deviated from the reference shear wave speed ratio between the hard and soft targets more than transaxial measurements. The smaller the target was, the closer the measurement (especially the longitudinal measurement) was to the background value, and the closer the shear wave speed ratio was to 1. For example, 9L‐D longitudinal measurement showed a ratio of 1.06 for the 4.05 mm target. These findings reflect the limitation of shear wave elastography function of these systems. Measurements of smaller targets on the order of 4 mm at 3 cm depth is challenging for either system.

There are several limitations of this study. First, there may be other clinical tasks related to SWE's utilization that clinical imaging physicists need to assess. We chose to report the AT in homogenous phantoms and comparison of target measurements in this study. These methods provided two general approaches which should be adaptable to other clinical tasks. For AT, we determined a test protocol based on our working environment, i.e., available equipment and personnel resources. Other approaches may also be feasible. For comparison of target measurements across vendors, there was only one relevant target depth in our commercial phantom. Phantoms allowing target measurements to be made at more depths might provide additional helpful information.

## 5 CONCLUSION

There are increasing SWE‐related tasks for clinical medical physicists, e.g., investigation and confirmation of proper SWE function for relevant clinical applications prior to clinical utilization. We reported approaches of clinical AT within 10 systems from one vendor, as well as comparison of target measurements across systems from two vendors. The test approaches and results reported should be helpful to other physicists needing to address these problems, and could also provide guidance for assessing system performance for other clinical SWE tasks.

## CONFLICT OF INTEREST

Pengfei Song and Shigao Chen receive royalties from GE Healthcare for the shear wave elastography function on the LOGIQ E9 scanner. The rest of the authors have no conflict of interest to declare.
